# Functional human cell-based vascularised cardiac tissue model for biomedical research and testing

**DOI:** 10.1038/s41598-022-17498-0

**Published:** 2022-08-05

**Authors:** Maria Koivisto, Tuomas A. Tolvanen, Tarja Toimela, Ilkka Miinalainen, Antti Kiviaho, Juha Kesseli, Matti Nykter, Lauri Eklund, Tuula Heinonen

**Affiliations:** 1 Faculty of Medicine and Health Technology, Tampere University, Arvo Ylpön katu 34, 33520 Tampere, Finland; 2 Biocenter Oulu Electron Microscopy Core Facility, University of Oulu, Oulu, Finland; 3 Computational Biology, Faculty of Medicine and Health Technology, Tampere University, Tampere, Finland; 4 Oulu Centre for Cell-Matrix Research, Faculty of Biochemistry and Molecular Medicine, Biocenter Oulu, University of Oulu, Oulu, Finland

**Keywords:** Tissue engineering, Drug safety, Drug screening, Toxicology, Gene expression, Stem cells

## Abstract

Cardiomyocytes derived from human induced pluripotent stem cells (hiPSC) are widely used in in vitro biomedical research and testing. However, fully matured, adult cardiomyocyte characteristics have not been achieved. To improve the maturity and physiological relevance of hiPSC-derived cardiomyocytes, we co-cultured them with preconstructed vascular-like networks to form a functional, human cell-based cardiac tissue model. The morphology and gene expression profiles indicated advanced maturation in the cardiac tissue model compared to those of a cardiomyocyte monoculture. The cardiac tissue model’s functionality was confirmed by measuring the effects of 32 compounds with multielectrode array and comparing results to human data. Our model predicted the cardiac effects with a predictive accuracy of 91%, sensitivity of 90% and specificity of 100%. The correlation between the effective concentration (EC50) and the reported clinical plasma concentrations was 0.952 (R^2^ = 0.905). The developed advanced human cell-based cardiac tissue model showed characteristics and functionality of human cardiac tissue enabling accurate transferability of gained in vitro data to human settings. The model is standardized and thus, it would be highly useful in biomedical research and cardiotoxicity testing.

## Introduction

The adverse cardiac effects of drugs are a major cause of drug attrition during drug development and post-approval market withdrawal^1,2^. The cardiotoxicity screening of new drugs typically includes in vitro non-myocyte-based cell assays and in vivo animal testing. The reliability of animal studies suffers from intrinsic electrophysiological differences between different species and humans^3^. The humanised cardiac ion channels in vitro models have been used in detecting compounds causing Torsades de Pointes arrythmia but their benefit for assessment of other cardiotoxic mechanisms as well as for efficacy screening is poor^4^. One reason is that drug-induced adverse cardiac effects can occur in multiple ion channels, structures and functions of the cardiovascular system^5^. Therefore, in vitro models comprising tissue type structure would be needed^4^. The use of human induced pluripotent stem cells (hiPSC)-derived cardiomyocytes in cardiotoxicity testing is proposed to be a more reliable screening platform for cardiac effects than the non-myocyte-based cell assay because hiPSC-derived cardiomyocytes resemble more accurately native human cardiomyocytes including the relevant ion channels^6^.

Even though effective protocols for cardiomyocyte differentiation in vitro have been developed^7,8^, maturation of the cardiomyocytes has remained a challenge. In their structure and function, the presently available hiPSC-derived cardiomyocytes more closely resemble embryonic or foetal cardiomyocytes than adult cardiomyocytes. These in vitro differentiated cardiomyocytes are typically circular, have irregularly arranged myofibrils and use glycolysis for ATP production, while adult cardiomyocytes are rod-shaped, have highly organized sarcomeres and utilize the oxidative phosphorylation of fatty acids for energy production^9,10^. For example, the sarcomere length and organization of hiPSC-derived cardiomyocytes is comparable to neonatal cardiomyocytes^11^. Immature and mature cardiomyocytes also have differences in, e.g., ion channel expression, calcium handling and electrophysiology^9,10^. Improving the maturity of the hiPSC-derived cardiomyocytes would increase their resemblance to the native adult myocardium and hence the predictivity of the results obtained using these test models^12^. Slight improvements have been obtained using approaches such as increased culture time, 3D culture methods and co-culture with non-myocytes^10,13^.

In the native human myocardium, the most abundant non-myocytes are fibroblasts, endothelial cells and peri-vascular cells. These cells have important functions in supporting normal heart homeostasis and in adaptation to pathological stimuli^14^. The role of the fibroblasts is to maintain the myocardial structure by secreting extracellular matrix components. They also mediate cardiomyocyte function by cell–cell interactions and paracrine factors^15^. Furthermore, fibroblasts can undergo phenotype conversion to proliferative myofibroblasts when augmentation of the matrix production is needed^16^. In the myocardium, endothelial cells form the capillaries that are essential for providing the cardiomyocytes with oxygen and nutrients in vivo. They also mediate cardiomyocyte spatial organization, contraction, survival and function. The physiological relevance of in vitro cardiac tissues could benefit from utilization of these complex and dynamic interactions between the different cell types as well as between the cells and the matrix^15^.

Previously we have shown that hASC-HUVEC co-culture produces a vascular network containing a lumen, and including different cell types such as endothelial cells, pericytes and smooth muscle cells^17,18^. In this paper, we present a cardiac tissue model that develops from a co-culture of a vascular-like network and hiPSC-derived cardiomyocytes under laboratory conditions to form a functional cardiac tissue model. We show the characteristics of the model on the structural and gene expression levels compared to a cardiomyocyte monoculture. The relevance of the model to predict human effects was verified by a set of known positive and negative substances, i.e., drugs with human data.

## Results

### Cardiomyocyte structural maturity improves when co-cultured with vascular-like networks

**Figure 1:**
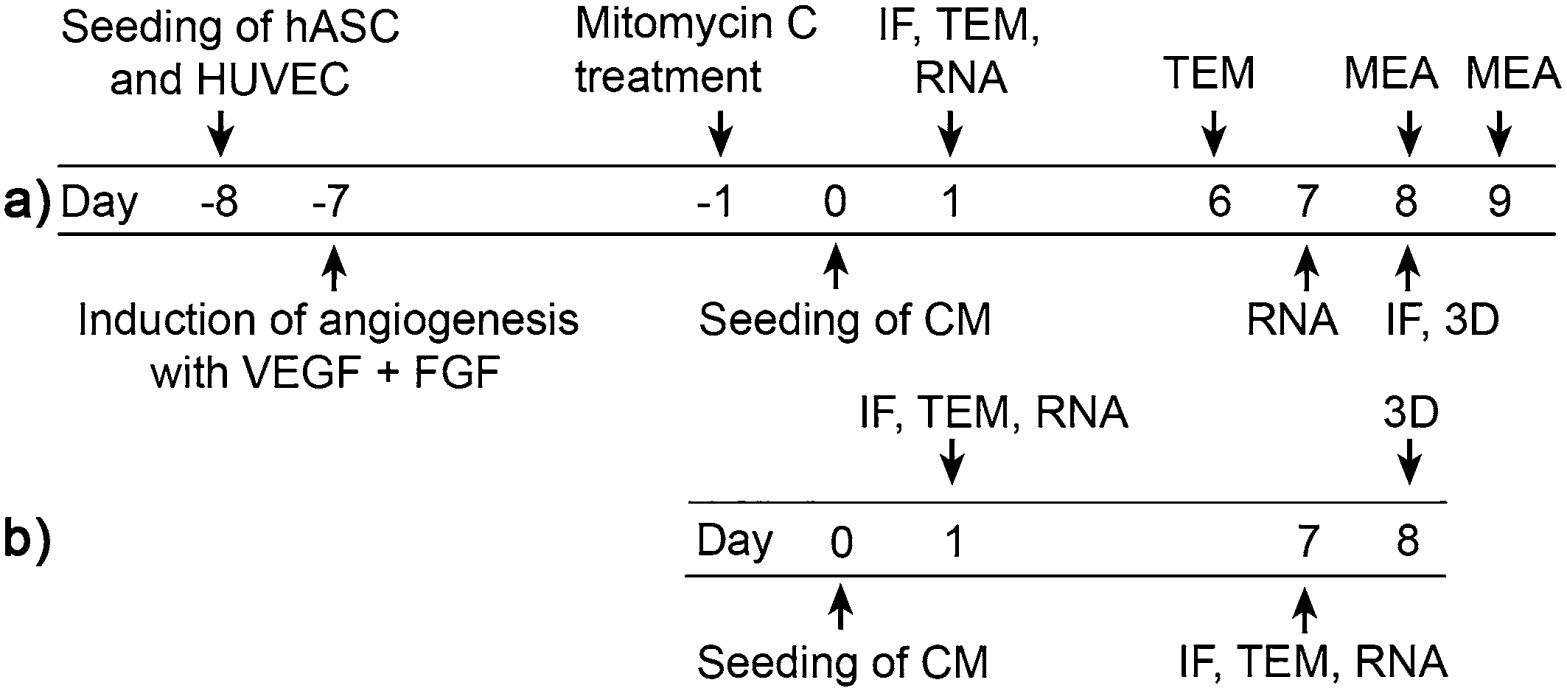
Cell culturing procedure. Cell culturing procedure for (**a**) the cardiac tissue model and (**b**) the cardiomyocyte monoculture. Time points for immunofluorescence (IF), immunofluorescence 3D images (3D), transmission electron microscopy (TEM), gene expression analyses (RNA) and electrophysiological measurements (MEA).

**Figure 2:**
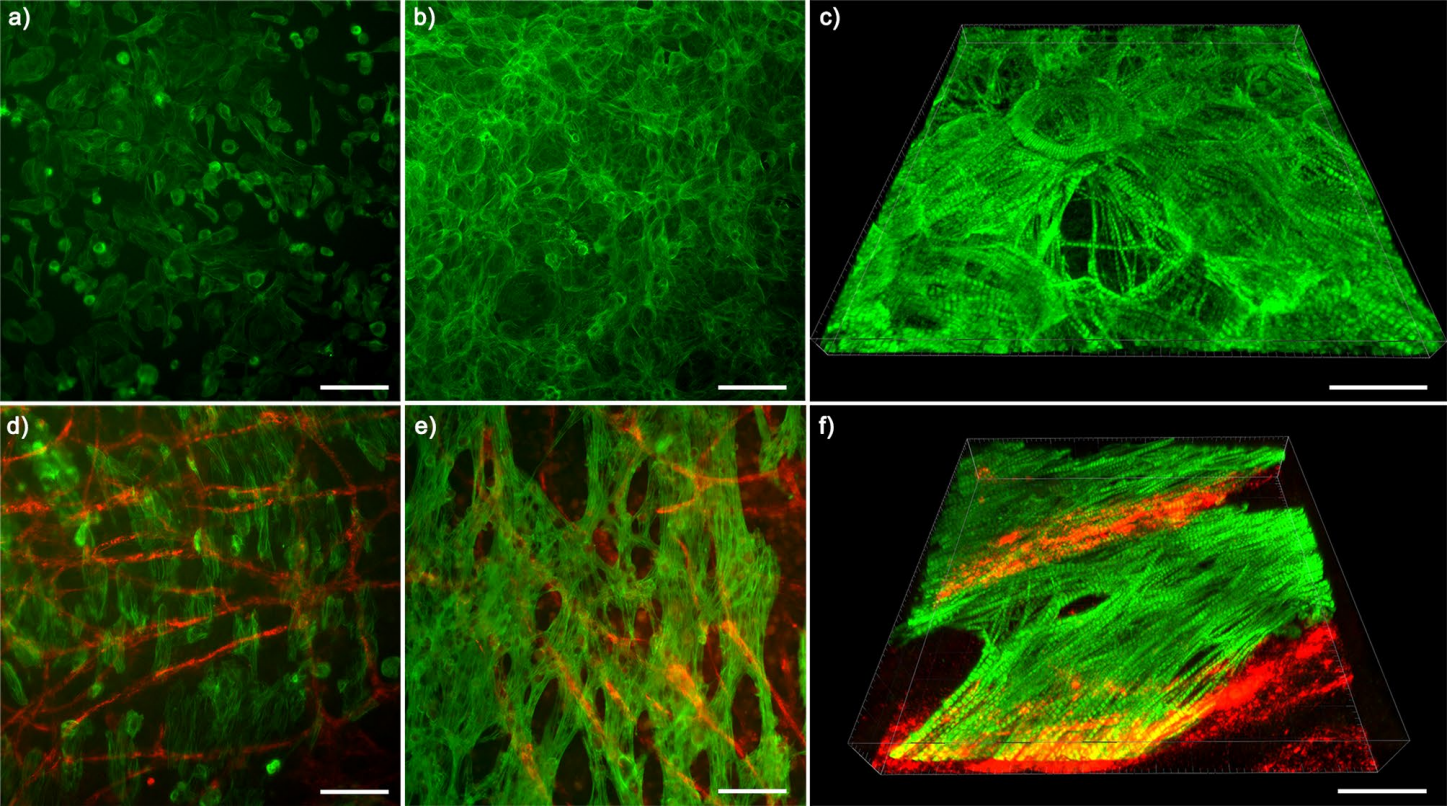
Immunofluorescence staining showed improved structural maturity of cardiomyocytes in cardiac tissue model compared to cardiomyocyte monoculture. (**a**) Immunofluorescence staining of the cardiomyocyte monoculture on day 1 and (**b**) day 7. (**c**) 3D-confocal microscopy image of the cardiomyocyte monoculture on day 8. (**d**) Immunofluorescence staining of the cardiac tissue model on day 1 and (**e**) day 8. (**f**) 3D-confocal microscopy image of the cardiac tissue model on day 8. Green: cardiac troponin T, red: von Willebrandt factor. Scale bar 100 µm (**a**,**b**, **d**, **e**), scale bar 30 µm (**c**, **f**). The experiment was repeated three times.

Cardiac tissue models and cardiomyocyte monocultures were established as presented in Fig. 1. The co-culturing of cardiomyocytes with the vascular-like networks affected the cardiomyocyte morphology remarkably, making it different from cardiomyocyte monocultures (Fig. 2). Already one day after the seeding, the shape of the cardiomyocytes was elongated in the cardiac tissue model, resembling their phenotype in cardiac tissues, whereas the cardiomyocytes in the monoculture were mainly circular. The difference further increased when the cardiomyocytes developed a connected network of aligned cardiomyocytes in the cardiac tissue model after one week. In the same time, the cardiomyocyte monoculture conserved the immature morphology and isotropic cell orientation. The orientation of myofibrils followed the cell shape and alignment. The myofibrils were highly oriented along the longitudinal direction of the cardiomyocytes in the cardiac tissue model (Fig. 2f), while in the monoculture, they oriented in multiple directions (Fig. 2c). The mean circular variance of cell orientation in cardiomyocyte monocultures was 0.90 (SD 0.06; N = 6) while the cardiomyocytes in cardiac tissue models were clearly more aligned with mean circular variance of 0.54 (SD 0.07; N = 15). The mean circular variance of myofilament orientation in a few neighbouring cardiomyocytes in the monoculture was 0.90 (SD 0.07; N = 3) and in the cardiac tissue model 0.61 (SD 0.08; N = 9). The improved alignment was statistically significant for both cell and myofilament orientation with *p* < 0.001 and *p* = 0.009, respectively.

**Figure 3:**
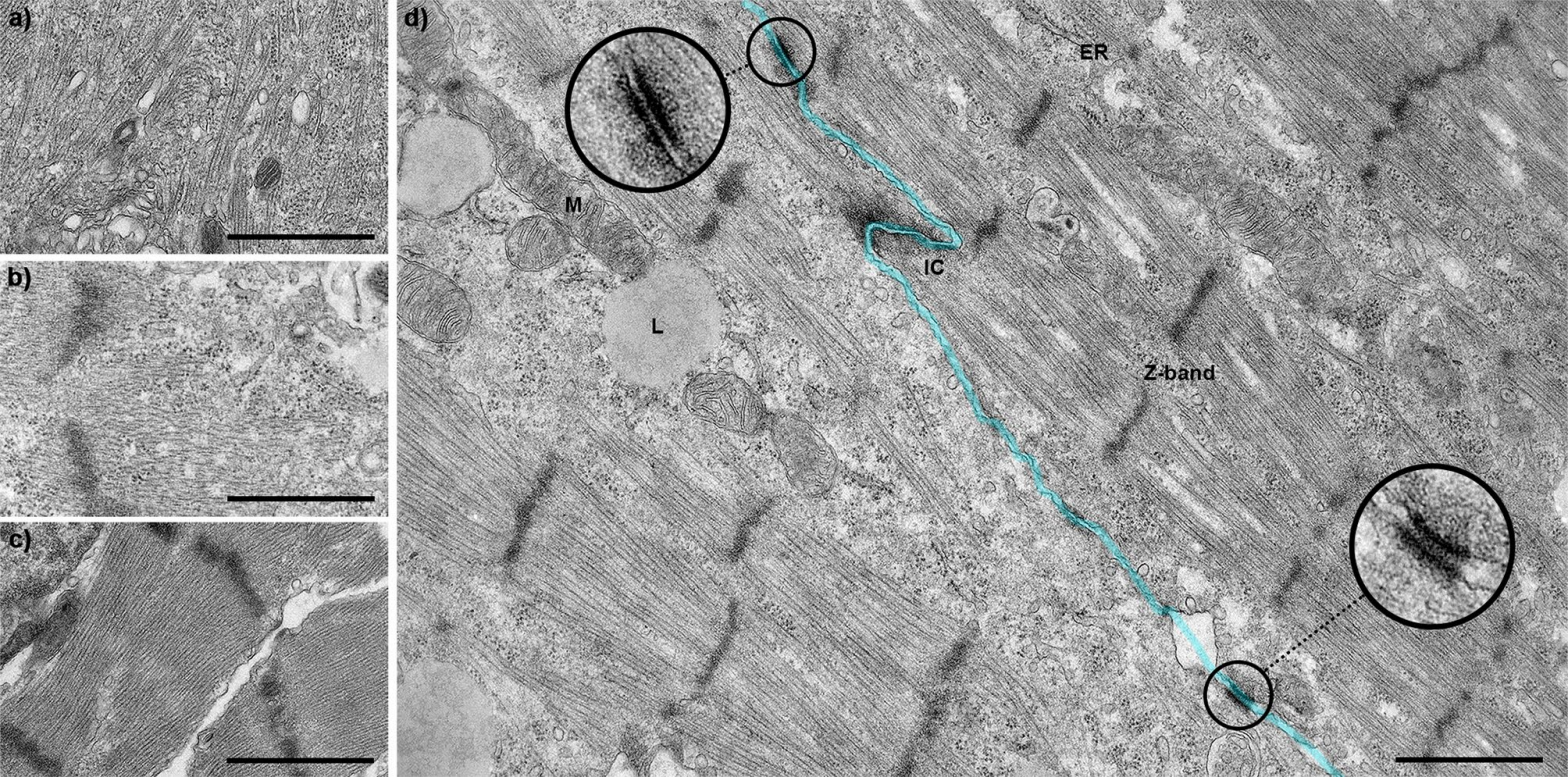
Transmission electron microscopy showed improved structural maturity of cardiomyocytes in cardiac tissue model compared to cardiomyocyte monoculture. Immature cardiomyocytes in (**a**) the cardiomyocyte monoculture and (**b**) the cardiac tissue model contain unorganized myofibrils on day 1. (**c**) Monocultured cardiomyocytes have developed organized sarcomeres by day 7. (**d**) Two adjacent cardiomyocytes with highly developed and aligned sarcomeres show intercalated discs (IC) and desmosome connections (circled and magnified) in the cardiac tissue model on day 6. Light blue indicates the line between two adjacent cardiomyocytes. ER = endoplasmic reticulum, M = mitochondria, L = lipid droplet. Scale bar 1 µm. The experiment was performed with one well from CM day 1, two parallel wells from CTM day 1, two parallel wells from CM day 7, and two parallel wells from CTM day 6.

Transmission electron microscopy (TEM) micrographs show that the monocultured cardiomyocytes were circular or slightly elongated on day 1. In the elongated cardiomyocytes, some aligned myofibrils were detected, and some of the myofibrils had arranged into sarcomere structures (Fig. 3a). Cell–cell connections had started to form. The cardiomyocytes in the cardiac tissue model were also immature on day 1 (Fig. 3b). After a week, the monocultured cardiomyocytes had developed well-formed sarcomeres (Fig. 3c). In the cardiac tissue model, sarcomeres with regular z-bands were highly aligned in adjacent cardiomyocytes six days after cardiomyocyte seeding (Fig. 3d). Intercalated discs connected cardiomyocytes, and transverse sections were located at the sarcomere z-bands. On the lateral side, desmosomes connected neighbouring cardiomyocytes, in contrast to the monocultured cardiomyocytes, which displayed immature intercalated discs and junctions. In the cardiac tissue model, the endoplasmic reticulum (ER) was also found near the sarcomere z-band. In the cardiac tissue model, the mitochondria appeared to be located in between and along the sarcomeres. Lipid droplets were located near the mitochondria and sarcomeres in the cardiomyocytes in the cardiac tissue model. Lipid droplets were not detected in the monocultured cardiomyocytes after one week. Although a large portion of the cardiomyocytes in the cardiac tissue models had developed many mature cardiomyocyte characteristics, some of the cells still displayed immature characteristics after one week. The number of myofilaments and mitochondria in the cytosol varied greatly between the cardiomyocytes. The cells that contained less of the regular myofilaments also had fewer intercalated disc connections to adjacent cardiomyocytes.

In addition to cardiomyocytes, numerous endothelial cells and myofibroblasts were found in the cardiac tissue model (Supplementary Fig. S1). Both cell types were present already one day after cardiomyocyte seeding. There were no significant changes in the gene expression levels of endothelial cell markers *PECAM1*, *CDH5*, and *VWF* between day 1 and 7 in the cardiac tissue model. However, these markers were clearly more expressed in the model compared to the monoculture at day 7. Endothelial cells are probably the remains of HUVECs but the myofibroblasts have developed in the model. Myofibroblasts can derive from endothelial cells via epithelial-mesenchymal transition or from mesenchymal cells via mesenchymal-epithelial transition. To reveal the origin of these myofibroblasts further studies are needed.

### Cardiomyocyte gene expression profile reached a more mature state in cardiac tissue model than in monocultured cardiomyocytes

**Figure 4:**
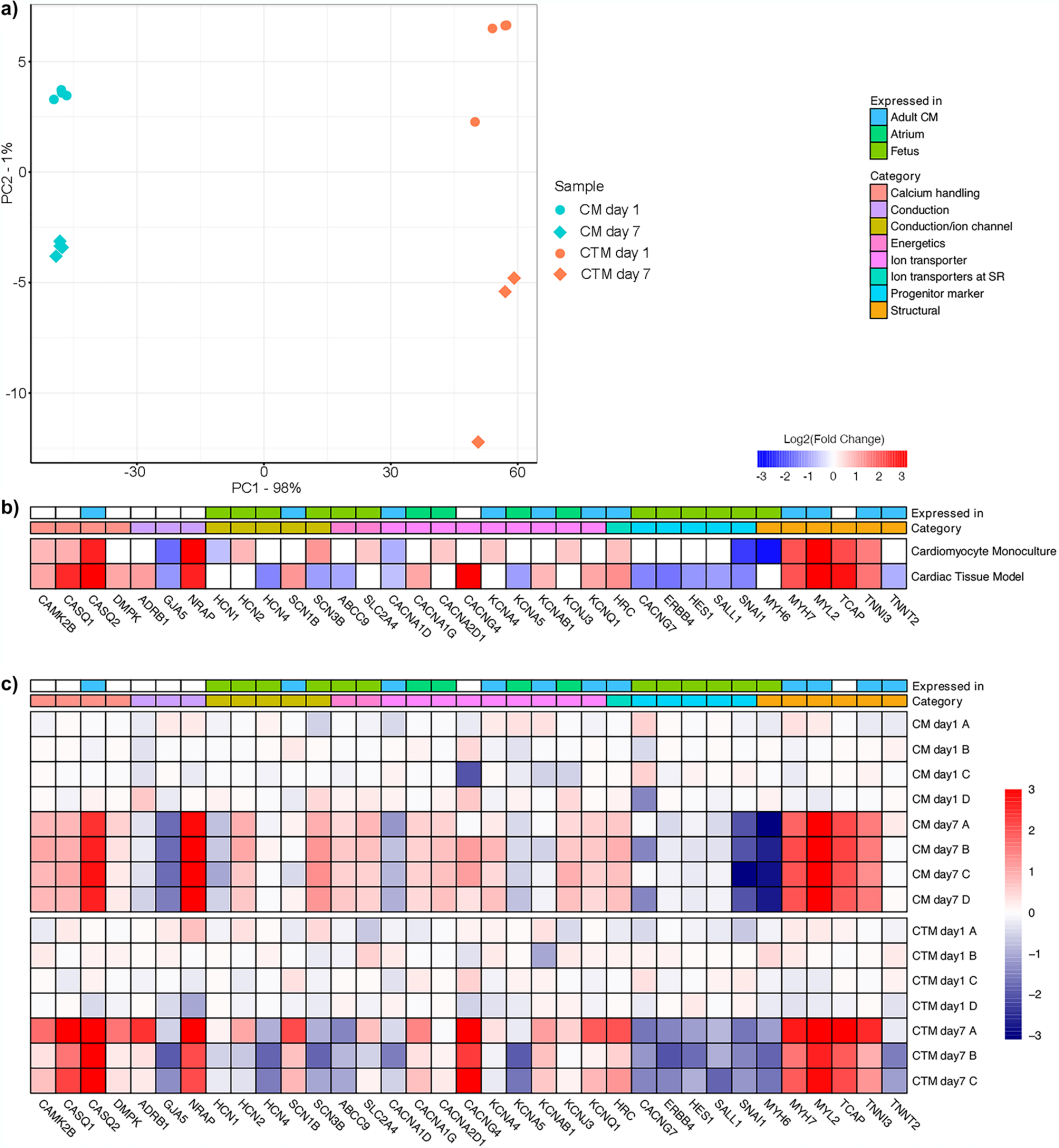
Cardiomyocyte gene expression profile reached a more mature state in cardiac tissue model than in monocultured cardiomyocytes. (**a**) PCA plot of cardiomyocyte monoculture (CM day 1 & CM day 7) and cardiac tissue model (CTM day 1 & CTM day 7) samples using top 500 most variable genes and variance stabilizing transformation (DESeq2). Numbers attached to the principal components correspond to the percentage of variance that is attributed to it. (**b**) Heatmap of the log2-transformed fold changes (day 1 to day 7) of the cardiomyocyte monoculture and cardiac tissue model samples. Pre-selected genes of interest are annotated according to category and typical site of expression. Values for genes with a statistically insignificant fold change (padj > 0.05) are not reported. (**c**) Heatmap of expression changes per gene for each sample. The DESeq2-normalized count of each gene was additionally normalized against the mean of the day 1 expression of each gene. Day 1 normalization was done separately for CM and CTM, followed by a log2 transformation. Genes and their annotations correspond to those of (**b**). Number of replicates from two repeats n = 4 for CTM day 1, CM day 1 and CM day 7; n = 3 for CTM day 7. The heatmaps were created in R 3.6.0 using pheatmap v1.0.12 (https://github.com/raivokolde/pheatmap).

To further confirm differences in the maturation of the monocultured cardiomyocytes and cardiac tissue model, we compared the gene expression patterns on day 1 and day 7 post cardiomyocyte seeding. Principal component analysis (PCA) of both the monocultured cardiomyocytes and cardiac tissue model show the day 1 and day 7 samples to be separated into their own clusters (Fig. 4a). Furthermore, we found that external RNA expression data from adult heart samples clustered closer to the cardiac tissue model samples than monocultured cardiomyocytes samples in the PCA space (Supplementary Fig. S2). Some 604 genes were significantly differentially expressed (*p* ≤ 0.05 and fold difference ≥ 1) in the monocultured cardiomyocyte samples, compared to 579 genes in the cardiac tissue model samples. In the monocultured cardiomyocyte samples, a positive log2 fold change (log2FC) was observed in 419 genes and a negative log2FC was observed in 185 genes. The corresponding counts for the cardiac tissue model samples were 237 positive log2FC genes and 342 negative log2FC genes (see the full lists of genes in Supplementary Table S1).

The RNA sequencing revealed that both the monocultured cardiomyocytes and cardiomyocytes in the cardiac tissue model were maturing during the seven days of culture (Supplementary Fig. S3, Supplementary Table S1), as the structural and ion channel coding genes were differentially expressed between days 1 and 7 (Fig. 4b). For example, the markers of myofibril maturation *MYH7*, *MYL2* and *TNNI3* were upregulated in both cardiac tissue model and cardiomyocyte monoculture. However, on day 7, the cardiac tissue model was more mature compared to the monocultured cardiomyocytes (Fig. 4c). The expression levels of sodium channel subunits *SCN1B*, predominantly expressed in the adult heart, and *SCN3B*, highly expressed in the embryonic heart and iPSC-derived cardiomyocytes^19^, shifted toward a more adult heart-like expression pattern in the cardiac tissue model, but not in the monocultured cardiomyocytes (Fig. 4c). Genes expressed in foetal heart and cardiomyocyte progenitor cells, such as *ABCC9*, *SALL1*, *HES1*, *CACNG7*, *SNAI1* and *ERBB4*, were significantly downregulated in the cardiac tissue model, but not in the monocultured cardiomyocytes (Fig. 4b). The monocultured cardiomyocytes also had an increased expression of *CACNA2D* and *KCNJ3*, genes coding the ion channels mainly expressed in the atrium. In contrast, *KCNA5*, an atrial ion channel, and *HCN4*, mainly expressed in the sinoatrial node, were significantly downregulated in the cardiac tissue model but not in the monocultured cardiomyocytes (Fig. 4b). Full lists of the differentially expressed genes and enriched GO terms are presented in the Supplementary Table S1.

### Cardiac tissue model yielded high predictivity in drug testing with high correlation to human data

**Figure 5:**
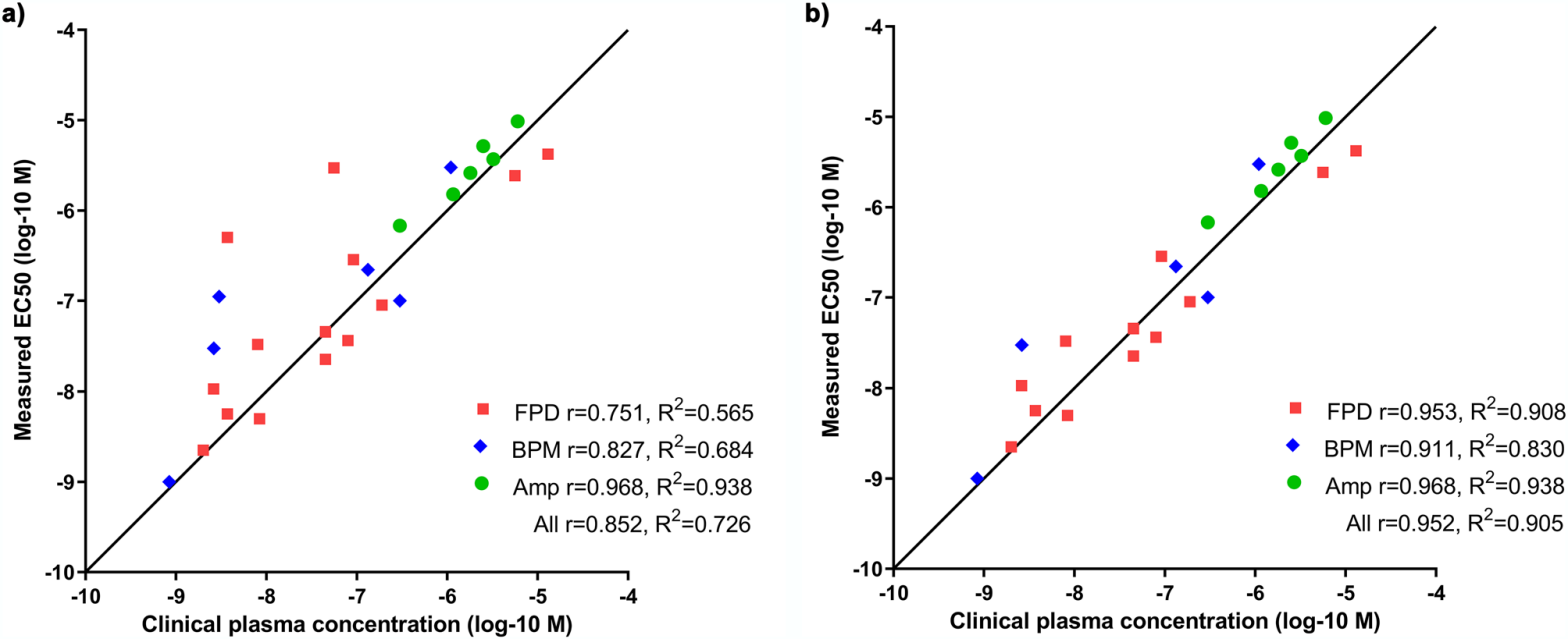
Cardiac tissue model yielded high predictivity in drug testing with high correlation to human data. Correlation between measured EC_50_ values and clinical plasma concentrations of (**a**) 26 study compounds (drugs) and (**b**) without alfuzosin, digoxin and tolterodine. Correlation scores for the groups are calculated using Pearson r. Goodness of fit is expressed as R^2^. Number of replicates n = 4–8 per repeat depending on MEA well format, repeated three times.

To investigate how the more mature morphology and gene expression profile affect electrophysiology, we measured the baseline electrophysiological characteristics of the cardiac tissue model with a microelectrode array (MEA). The baseline field potential duration (FPD) was 441 ms (SD 42 ms) and the beating frequency was 45 (SD 4) beats per minute (BPM). To test the applicability of the cardiac tissue model to identify cardiac effects, we exposed the model to 29 substances with known effects on cardiac function and three negative controls known to have no effect (see Table 1 for the compounds and their known mechanisms). We used the multiwell format of MEA, which allows medium throughput capacity with a 96-well plate. As the selected test substances covered many different mechanisms of action, we analysed beating frequency and sodium spike amplitude in addition to the regularly analysed FPD. Table 1 presents the measured EC_50_ values for each parameter and Δ_20_ value, which represents the first concentration that caused a change over 20% to the parameter compared to the baseline. The biological significance level of 20% change was chosen based on preceding literature^20–22^. We found that 26 out of the 29 positive reference compounds presented activity characteristics that have been previously reported in literature. The concentration-dependent effects of these 26 study compounds are presented in Supplementary Fig. S4. None of the three negative controls (Acetyl salicylic acid, Hexylresorcinol and Ibuprofen) showed signs of cardio activity or toxicity. This yielded a predictive accuracy of 91%, sensitivity of 90% and specificity of 100%. Compounds that were not recognized or expected to cause the clinically known effect in our test setting were doxorubicin, levosimendan and pentamidine. The correlation of the measured EC_50_ values to the reported clinical plasma concentrations was for all the compounds 0.852, with R^2^ = 0.725 (Fig. 5a). With epinephrine, isoprenaline and propranolol the Δ_20_ value was used for the calculation of the correlations as the clinical concentrations of these drugs alter the beating frequency about 20%. Our model gave excellent prediction for the drugs with the amplitude as the endpoint, with a correlation of 0.968 (R^2^ = 0.938). The substances with an over tenfold difference between the measured EC_50_ value and the clinically relevant plasma concentration were alfuzosin, digoxin, and tolterodine. The detected effects of these three drugs are clinically observed only in overdose and thus can be excluded from the correlation, leading to a correlation of 0.952, with R^2^ = 0.905 (Fig. 5b).

**Table 1:** Field potential duration (FPD), beats per minute (BPM), and Na^+^ peak amplitude (Amp) Δ_20_ and EC_50_ values of all test compounds and therapeutic concentration of the compound. The symbol ↑ indicates an increase and the symbol ↓ indicates a decrease in the measured parameter. The reported concentrations from our model are the total (nominal) concentrations, and the therapeutic concentrations from the literature are the free plasma concentrations.

Compound	Known mechanism	Action	End point	FPD Δ_20_ (nM)	FPD EC_50_ (nM)	BPM Δ_20_ (nM)	BPM EC_50_ (nM)	Amp Δ_20_ (nM)	Amp EC_50_ (nM)	Therapeutic concentration (nM)
Alfuzosin	QT prolonging	Na + channel activator alfa-AR antagonist	FPD	↑300	↑3,228	↓10,000	↓6,775	↓10,000	↓6,528	56^21^
Haloperidol	QT prolonging	K + channel blocker	FPD	↑3	↑22.71	↓10	↓45.21	↓30	↓103.1	45^60^
Astemizole	QT prolonging	hERG blocker	FPD	↑30	↑33.12	↓100	↓1,043	↓100	↓1,274	8^21^
Cisapride	QT prolonging	hERG blocker	FPD	↑3	↑10.58	↓300	↓3,694	↓3,000	↓4,432	2.5^29^
Dofetilide	QT prolonging	hERG blocker	FPD	↑1	↑1.638	↓3	↓2.085	↓10	↓3.491	2^60^
E-4031	QT prolonging	hERG blocker	FPD	↑3	↑5.001	↓30	↓57.38	↓30	↓50.68	8.4^60^
Moxifloxasin	QT prolonging	hERG blocker	FPD	↑10,000	↑2,431	–	–	–	–	5600^60^
Sotalol	QT prolonging	hERG blocker	FPD	↑1,000	↑4,209	↓30,000	↓6,051	–	–	13,000^60^
Thioridazine	QT prolonging	hERG blocker	FPD	↑300	↑45.43	↓3,000	↓2,239	↓1,000	↓1,854	45^60^
Tolterodine	QT prolonging	hERG blocker	FPD	↑100	↑502.8	↓3,000	↓4,593	↓3,000	↓3,046	5
Pimozide	QT prolonging	hERG and Ca2 + channel blocker	FPD	↑10	↑5.643	↓30	↓1,466	↓100	↓307	3.7^61^
Isradipine	QT shortening	L-type Ca2 + channel blocker	FPD	↓10	↓36.57	↑10	↑109.8	↓3,000	↓3,314	80^61^
Nifedipine	QT shortening	L-type Ca2 + channel blocker	FPD	↓30	↓89.56	↑30	↑296.9	↓10,000	↓7,797	190^60^
Verapamil	QT shortening	L-type Ca2 + channel blocker	FPD	↓100	↓285.3	↑10	↑103.6	↓3,000	↓2,633	92^60^
Digoxin	Positive inotropic	Na/K ATPase blocker	BPM	–	–	↑100	↑111.2	–	–	2.8^61^
Dopamine	Positive inotropic	dopamine receptor, AR-receptor agonist	BPM	↓300	↓109.3	↑100	↑119.4	–	–	300 (20% increase)
Epinephrine	Positive inotropic	AR agonist	BPM	–	–	↑30	↑183.5	–	–	2.64^62^
Isoprenaline	Positive inotropic	AR agonist	BPM	–	–	↑1	↑31.53	↑100	↑33.65	0.84^63^
Doxazosin	Negative inotropic	alfa1-AR blocker	BPM	↑300	↑29.17	↓100	↓220.6	↓300	↓288.5	133^61^
Propranolol	Negative inotropic	AR blocker	BPM	–	–	↓3,000	↓6,902	↓3,000	↓7,606	1095^64^
Flecainide	Negative inotropic	Na + channel blocker	Amp	↑300	↑162.9	↓1,000	↓1,297	↓1,000	↓1,516	1160^60^
Lidocaine	Negative inotropic	Na + channel blocker	Amp	–	–	↓3,000	↓4,980	↓3,000	↓5,157	2500^29^
Quinidine	QT prolonging	Na + channel blocker	Amp	↑300	↑505.9	↓1,000	↓5,061	↓1,000	↓3,701	3240^60^
Ranolazine	Negative inotropic, QT prolonging	Na + channel blocker, hERG blocker	Amp	–	–	↓30,000	↓8,579	↓10,000	↓9,709	6010^21^
Terfenadine	QT prolonging	hERG blocker, multi-channel blocker	Amp	↓1,000	↓900.2	↓1,000	↓1,419	↓300	↓680.4	300^21^
Terodiline	QT prolonging	hERG blocker, Ca2 + channel blocker	Amp	–	–	↓3,000	↓3,805	↓1,000	↓2,607	1800^60^
Acetyl salicylic acid	No effect on heart function		–	–	–	–	–	–	–	–
Hexylresorcinol	No effect on heart function		–	–	–	–	–	–	–	–
Ibuprofen	No effect on heart function		–	–	–	↑30,000	–	–	–	–
Doxorubicin	apoptotic	ROS?	–	–	–	–	–	–	–	
Levosimendan	Positive inotropic	Ca2 + sensitizer	–	–	–	–	–	–	–	129^21^
Pentamidine	QT prolonging	Effects hERG channel transport	–	–	–	↑30,000	–	↑300	–	190^61^

## Discussion

The results of the structural characterization together with the RNA sequencing imply that the cardiomyocytes develop further when cultured with in vitro constructed tubular network. The vascular-like network that was included in this cardiac tissue model has been characterized previously^17,18^. The hASC-HUVEC co-culture forms a tubular network of endothelial cells with perivascular and smooth muscle cells in six days^17^. In the cardiac tissue model, the endothelial cells and myofibroblasts were found one day after cardiomyocyte seeding. These cell types are normally present in the healthy myocardium^14^, except the myofibroblasts, whose presence typically indicates remodeling of the extracellular matrix^23^.

A previously reported in vitro cardiac tissue, cultured with supporting dermal fibroblasts within an artificial scaffold and with electrical stimulation, has been shown to mimic well the cardiomyocytes of an adult heart^24^. Our method to produce a mature cardiac tissue model is technically less challenging and composed of commercially available cell lines. In our model, the co-cultured cells develop into a cardiac tissue-mimicking construct in vitro without artificial scaffolds or external stimulation. This model presents reliable and cost-effective tool for screening acute cardiac effects of drugs.

Morphological differences between the cardiac tissue model and monocultured cardiomyocytes indicate that the crosstalk between the different cell types and contact with the extracellular matrix are crucial for the maturation of cardiomyocytes. This can be mediated by endothelial cell-cardiomyocyte paracrine signalling which plays an important role in cardiac tissue growth and remodelling^25^, and by cardiomyocyte-extracellular matrix connections which induce the rod-shaped phenotype of cardiomyocytes during development and contribute the structural integrity of the heart^26^. In the native myocardium, each cardiomyocyte is located next to at least one capillary, which ensures the adequate oxygen and nutrient supply in vivo but also enables vesicle transport and local communication between cardiomyocytes and endothelial cells^27^. Moreover, both cardiomyocytes and endothelial cells express connexins, that form gap junctions which may play a role in the cardiomyocyte—endothelial cell interaction^25,28^. The presence of lipid droplets next to the sarcomeres and mitochondria in the cardiomyocytes in our cardiac tissue model (Fig. 3d) suggests the transfer of energy metabolism from foetal glycolysis to the oxidation of phospholipids^24^. Moreover, the improved organization and alignment of cardiomyocytes (Fig. 2e) and sarcomeres (Fig. 3d) in the cardiac tissue model indicates advanced maturation compared to the cardiomyocyte monoculture (Fig. 2b)^10^. The sarcomeres of mature adult cardiomyocytes are longer, approximately 1.7–2.2 µm depending on contraction stage, than the sarcomeres of immature cardiomyocytes^11,13^. We did not notice longer sarcomeres in cardiac tissue models compared to cardiomyocyte monocultures. However, for a reliable comparison of the sarcomere lengths in the cardiac tissue model and cardiomyocyte monoculture we would have needed a greater number of images in this study.

As the cardiomyocytes used for the RNA sequencing were from two differentiation batches, the batches caused some variance between them (Fig. 4c). The results show that both the monocultured cardiomyocytes and cardiomyocytes in the cardiac tissue model matured during the seven days of the culture. However, the monocultured cardiomyocytes were still expressing more genes present in the cardiac progenitor cells and in the atrium on day 7 compared to the cardiac tissue model (Fig. 4b). Interestingly, 18 of the 131 GO terms enriched in the cardiac tissue samples contain ‘heart’ or ‘cardiac’ in their name, compared to only 2 out of the 91 for the monocultured cardiomyocyte samples (Supplementary Table S1). This suggests that the cardiomyocytes in our cardiac tissue model are more mature and more of the homogenously ventricular type of cardiomyocytes.

Monocultured iCell cardiomyocytes have been previously reported to have an FPD of 468 ms (SD 42) and a BPM of 38 (SD 2.5)^29^, while in humans, QT times ≤ 420 ms and a heart rate of 60 BPM are considered normal. In our cardiac tissue model, the FPD of 441 ms (SD 42) and beat rate of 45 BPM (SD 4) were closer to the physiological values of the human heart compared to the monocultured cardiomyocytes.

The high goodness of fit (R^2^ = 0.938) and correlation (r = 0.963) between observed IC50 values and the clinical plasma concentration of the sodium channel blockers are likely due to the more adult-like expression pattern of the sodium channels (SCN1B vs SCN3B) in our cardiac tissue model. SCN1B is predominantly expressed in adult heart and SCN3B in foetal heart^19^. Culturing cardiomyocytes with supporting dermal fibroblasts within an artificial scaffold and with electrical stimulation was not sufficient to induce SCN1B expression^24^. Thus, it seems that the addition of stromal cells (hASC), which can differentiate into multiple different cell types and create a more natural environment, enhances the maturation of the cardiomyocytes. The IC50 values for flecainide, lidocaine and quinidine were closer to the therapeutic concentrations than previously reported IC50 values from hiPSC-derived cardiomyocytes^30^.

Of the QT-prolonging compounds, the measured FPD-prolonging EC50 values of alfuzosin and tolterodine were more than tenfold above the clinical plasma concentrations. At high doses (100 nM), alfuzosin is reported to cause a mild QT increase in patients and significantly prolong the QT interval in vitro at a concentration of 300 nM^31^. At 300 nM, we detected a statistically significant 20% increase in FPD. Tolterodine does not prolong the QT interval in clinical use^32^, and the QT-prolonging effect has been reported only with a very high concentration in vitro (corresponding to a 340 nM concentration in vivo). These QT prolongations are identified adverse effects for both drugs, and they have been reported with plasma concentrations above the normal clinical plasma concentrations, which is supported by our data. This also suggests that our model could be used to predict safety margins for the drugs. In addition, the EC/IC50 values from our model were closer to the therapeutic concentrations than previously reported values for e.g., nifedipine^33,34^, isradipine^35^, sotalol^34^, and verapamil^35^. For other compounds, such as E-4031^33,35^, the previously reported EC/IC50 values were already close to the therapeutic concentrations.

Of the compounds affecting heart rate, the measured EC50 values for digoxin and propranolol differed from their clinical plasma concentrations more than tenfold. Tachycardia is an adverse effect of a mild overdose of digoxin. Thus, the higher concentration needed in our model for BPM changes compared to the situation in the clinical setting for digoxin is likely to be explained by the slow distribution of digoxin shown in humans^36^. The EC50 values were closer to the therapeutic concentrations than previously reported values for e.g., isoprenaline^34^.

Doxorubicin is reported not to affect FPD or beat rate during short exposures, but to increase the apoptosis of hiPSC-derived cardiomyocytes after two days of exposure^37^. As we expected, we could not detect any doxorubicin-induced changes to FPD, BPM or amplitude in our short-term experiments. Doxorubicin appears not to affect the electrophysiology of cardiomyocytes during short exposures up to 2 h. Levosimendan is a positive inotrope which increases the beating force of the cardiomyocytes by binding to troponin C in a calcium-dependent manner^38^. Using MEA, it is not possible to measure beating force. Therefore, we did not expect levosimendan to show any effect in MEA. Pentamidine has been reported to block the transport of hERG channels to the cardiomyocyte plasma membrane^39^. As the half-life of hERG channels is 2.8 h^40^, we could not detect any significant FPD prolongation in our test setting where pentamidine concentration exceeds the clinical plasma concentration only for 50 min.

Terfenadine is clinically shown to prolong QT time. However, we detected terfenadine-induced FPD shortening. We also detected a non-significant 10% increase in FPD, similarly as reported by Mehta et al.^41^, before the shortening of FPD initiated. Terfenadine acts as an unspecific multi-channel blocker and not only as a hERG blocker^42^, with reported IC50 to calcium channels at 185 nM^43^, hERG at 350 nM^44^ and sodium channels at 930 nM in canine atrial myocytes^45^. These multi-channel effects might explain the unexpected results obtained with terfenadine. In our model, the blocking of sodium channels appears to be the major effect of terfenadine, which is also in line with the previously reported results of terfenadine in monocultured cardiomyocytes^41^.

We did not detect the expected FPD prolongation of terodiline. However, we could detect its cardiotoxicity based on its effects on beating amplitude. Terodiline has two optical isomers, R- and S-terodiline, which were both present in the racemic terodiline used in our experiments. R-terodiline shows high anticholinergic properties, responsible for the prolongation of FPD^46^, whereas S-terodiline shows calcium antagonist properties^47^. It is likely that the FPD shortening effect of calcium antagonist S-terodiline concealed the FPD prolongation effect of R-terodiline. The reported toxic plasma concentration for terodiline in humans is 2.1 µM^48^, which is very close to the amplitude EC50 concentration (2.6 µM) detected in our experiments. This together with the results from terfenadine emphasizes the importance of monitoring not only the FPD changes but also BPM and amplitude, which are measured by default while using MEA, to better detect the cardiotoxic effects of the compounds.

This cardiac tissue model aims to mimic human heart tissue. Despite improvements in the cardiac tissue maturation, it does not fully recapitulate the adult state of the human heart tissue. The cardiac tissue model also does not recapitulate the structure of the whole human heart. For example, the tissue model does not have connections with nerve stimulation. Furthermore, the model measures only the direct effects of the parent drugs as the cardiomyocytes present only weak drug metabolism. MEA measurements are useful to detect many types of drug effects. However, certain drugs mechanisms of actions remain undetected with this method. For example, MEA cannot detect changes in the beating force of the cardiomyocytes, so the inotropic effects are missed if they do not affect the beating frequency e.g., levosimendan. Moreover, only acute short-term effects of the compounds were measured in this study. For effects requiring longer time to develop, culture and exposure times could be extended. Cardioactive drugs that require longer time to affect e.g., pentamidine, were not recognized. In addition, the balance between positive (29) and negative (3) test substances could impact the determination of the sensitivity, specificity and accuracy values in this study.

In this research, we have shown that hiPSC-derived cardiomyocytes cultured together with a vascular-like network form a functional cardiac tissue-mimicking construct in vitro. The morphology and gene expression levels of our cardiac tissue model show the characteristics of an adult heart, and the maturation of the cardiomyocytes is more advanced when compared to cardiomyocyte monocultures. The functional characterization with the positive and negative reference compounds compared with human data shows that the data gained from our model are transferable to the clinical setting for the evaluation of cardiac effects, including acute cardiotoxicity.

## Methods

### Human cell-based cardiac tissue model

This study conforms to the principles outlined in the Declaration of Helsinki. The use of human adipose stromal cells (hASCs) obtained from surgical operations and the use of human umbilical vein endothelial cells (HUVECs) from scheduled caesarean sections was approved by the Ethics Committee of Pirkanmaa Hospital District (permit numbers R15161 and R15033, respectively). Informed consent was received from the tissue donors prior the study. The hASCs and HUVECs were isolated and propagated as previously described^17,49^. They were maintained in a humidified incubator at + 37 °C, 5% CO_2_, and the medium was refreshed every 2–3 days. The hASC lines were confirmed CD90, CD73 and CD105 positive by flow cytometry as previously described^49^, and passage 2 was used in cultures. The HUVEC lines passed a tube formation test as previously described^49^, and passage 4 was used in cultures. The hASC and HUVEC lines were tested negative for mycoplasma contamination.

To improve the long-term attachment of the cells, a thin fibrin coating was applied to the cell culture wells by mixing a 1:1 solution containing 5.5 mg/ml fibrinogen (Sigma Aldrich, F3879) with 38 µg/ml aprotinin (Sigma Aldrich, A1153) and a solution of 2.75 UN/ml thrombin (Sigma Aldrich, T7009). Excess solution was removed prior to incubation for 45 min at + 37 °C.

To produce the vascular-like networks, hASCs were seeded in the fibrin-coated wells at 20,000 cells/cm^2^ and HUVECs were seeded on top of them at 4000 cells/cm^2^ 1–4 h later in EBM-2 with EGM-2 SingleQuots supplements (Lonza, CC-3162) as previously described^49^. The next day, angiogenesis stimulation was initiated using a serum-free stimulation medium (SFSM) consisting of DMEM/F12, 2.56 mM L-glutamine, 0.1 nM 3,3′,5-Triiodo-L-thyronine sodium salt, ITS™ Premix: 1.15 μM: 6.65 μg/ml insulin, 6.65 μg/ml Transferrin, 6.65 ng/ml selenious acid, 1% Bovine serum albumin, 2.8 mM Sodium pyruvate, 200 μg/ml Ascorbic acid, 0.5 μg/ml Heparin, 2 μg/ml Hydrocortisone, 10 ng/ml VEGF, and 1 ng/ml FGF-β as described earlier^18^. On day 4, the SFSM was refreshed. After seven days of co-culture, the cells were treated with 1 µg/ml Mitomycin C (Millipore, 47,589) for 1.5 h to impair their mitotic activity. Co-cultures were washed three times with warm PBS before the administration of fresh SFSM. On the next day, hiPSC-derived cardiomyocytes (iCell^2^, Cellular Dynamics) were seeded on top of the vascular-like networks at 156,000 cells/cm^2^ in iCell Cardiomyocytes Plating Medium (Cellular Dynamics, M1001). The plating medium was replaced with 1:1 SFSM and iCell Cardiomyocytes Maintenance Medium (Cellular Dynamics, M1003) 4 h later. The cells were maintained in a humidified incubator at + 37 °C, 5% CO_2_, and the medium was refreshed every second day.

### Cardiomyocyte monoculture

Cardiomyocyte monocultures were established by seeding iCell^2^ cardiomyocytes in fibrin-coated wells at 156,000 cells/cm^2^ in Cardiomyocytes Plating Medium. The medium was replaced by iCell Cardiomyocytes Maintenance Medium 4 h later. Cardiomyocytes were maintained in a humidified incubator at + 37 °C, 5% CO_2_, and the medium was refreshed every second day.

### Immunocytochemistry

For immunocytochemistry, the cells were seeded in chambered coverslips. The cells were fixed with 70% ethanol or 4% formaldehyde (3D images) for 20 min on day 1 and day 7 or 8 after cardiomyocyte seeding (time points are presented in Fig. 1). The cells were stained with monoclonal mouse anti-Troponin T antibody (1:100, Invitrogen, MA5-12,960, lot TK2667027) and polyclonal rabbit anti-von Willebrand factor (1:100, Dako, A0082, lot 20,030,046 and 20,067,357) at + 4 °C overnight. Secondary antibodies anti-mouse IgG Alexa Fluor 488 (Invitrogen, A21202, lot 2,018,296) and anti-rabbit IgG Alexa Fluor 594 (Invitrogen, A21207, lot 2,066,086) or FITC (Sigma Aldrich, F4143, lot 018M4797V and 045M4881V) and TRITC (Sigma Aldrich, T6778, lot 036M4785V) (3D images) were incubated for 45 min at RT. The cells were imaged using a Nikon Eclipse TiS with a 20 × objective and a Zeiss Laser Scanning Confocal Microscope LSCM 780 with a 63 × objective (3D images). Images were prepared with Photoshop CC (Adobe) except the 3D images which were prepared with Imaris software (Bitplane, Zürich, Switzerland). Cardiomyocyte and myofilament orientation analyses was performed using CytoSpectre 1.2 software (http://www.tut.fi/cytospectre)^50^. The software calculates the orientation of image components based on spectral analysis. The variance of image structure orientation is described as circular variance on a scale from 0 (perfect alignment) to 1 (perfect isotropy). In the software, mixed component mode was used, and spectral resolution/noise was set to balanced. Images taken with 20 × or 25 × objective were used for cardiomyocyte orientation analysis, and confocal images taken with 63 × objective were used for myofilament orientation analysis.

### Electron microscopy

Cardiac tissue constructs and cardiomyocyte monocultures were fixed for 60 min with 4% paraformaldehyde + 1% glutaraldehyde in 0.1 M phosphate buffer on day 1 and day 6 (cardiac tissue model) or day 7 (cardiomyocyte monoculture) after cardiomyocyte seeding. The cells were carefully removed from the dishes and pelleted. Fixed cell pellets were postfixed in 1% osmiumtetroxide, dehydrated in acetone and embedded in Epon LX 112 (Ladd Research Industries). Thin Sects. (70 nm) were cut with a Leica Ultracut UCT ultramicrotome, stained in uranyl acetate and lead citrate, and examined in a Tecnai G2 Spirit transmission electron microscope (FEI EuropeImages were captured by a Quemesa CCD camera and analysed using iTEM software (Olympus Soft Imaging Solutions GMBH).

### Gene expression

Gene expression was analysed by RNA sequencing from four replicate cultures of the cardiac tissue model from day 1 and from three replicate cultures from day 7, and from four samples per time point for the cardiomyocyte monoculture. The total RNA was collected using a PureLink RNA Mini Kit (Life Technologies, 12183018A) following the manufacturer’s protocol on days 1 and 7 after cardiomyocyte seeding. The quality of the mRNA samples was verified with Agilent 2100 Bioanalyzer and RNA sequencing, and the initial bioinformatics were performed by Novogene CO Ltd. (Beijing, China). RNA sequencing included cDNA library preparation and sequencing (20 M clean reads/sample) using Illumina PE150 (Q30 ≥ 80%) equipment.

Initial quality control was done using FastQC^51^, after which raw reads were mapped to GRCh38 (GENCODE comprehensive annotation v29) using STAR v2.7.1^52^. The resulting Binary Alignment Map (.bam) files were sorted and indexed using SAMtools v1.9^53^. Gene counts were accessed using featureCounts v2.0.1^54^ on hg38 (GENCODE basic annotation v34) and filtered for protein coding genes using biomaRt v2.40.5^55^.

To determine suitable replicates for downstream expression analysis, each sample was plotted on a two-dimensional graph using dimensionality reduction through principal components. Differential expression analysis was performed using DESeq2 v1.24.0^56^. Day-specific differences in gene expression were determined using the “contrast” function on DESeq2. Significant differentially expressed genes were filtered using a Benjamini–Hochberg adjusted p-value (BH *p*-value) of less than 0.05 and an absolute log2-fold-change of more than 1 as thresholds. Enrichment analysis was performed on the resulting gene lists using the g:GOSt functional profiling tool^57^ and a significance threshold of a BH p-value less than 0.01. Gene Ontology biological processes (GO:BP, ENSEMBL 99 release 2020–01-01) excluding evidence Inferred from Electronic Annotation (IEA) and KEGG pathways (KEGG FTP release 2020–02-03) were used as databases^58^. If a gene symbol in the query matched multiple ENSEMBL ids, the one with the most GO annotations was used. The annotated genes for the heatmaps were selected from the DEGs (*p* ≤ 0.05) found under the significant GO terms including words “heart” and “cardiac” and from previous publications^10,13,24^. The genes were chosen based on their expression primarily in cardiomyocytes^10,13,24^.

### Preparation of stock solutions used in electrophysiology

Hydrophobic compounds were solubilized in DMSO as 10 mM stocks. The water-soluble compounds were solubilized in distilled water as 1 mM stocks. These stocks were diluted to the exposure medium (DMEM/F12 + 2.56 mM L-glutamine, 0.1 nM 3,3′,5-Triiodo-L-thyronine sodium salt, ITS™ Premix: 1.15 μM: 6.65 μg/ml insulin, 6.65 μg/ml Transferrin, 6.65 ng/ml selenious acid, 1% Bovine serum albumin, 2.8 mM Sodium pyruvate).

### Electrophysiology

For the electrophysiological measurements, the cardiac tissue models were cultured in 24-well or 96-well multielectrode array (MEA) plates (Multi Channel Systems, 24W700/100F-288 or 96W700/100F-288). Extracellular field potentials were recorded using a Multiwell-MEA-System (Multi Channel Systems) with a 20 kHz sampling frequency on days 8–9 after cardiomyocyte seeding. The temperature was set at 37 °C and the cells were supplied with 5% CO_2_ during the measurements. Two-minute recordings were obtained as spontaneous baseline beating, after which the cardiac tissue models were exposed to a ½ logarithmic cumulative concentration series spanning from 300 to 30 µM of the test compounds. With 24-well plates four replicate wells and with 96-well plates eight replicate wells were used for each compound. The wash-in period between administration of the test compounds and 2-min recording was 8 min. The field potentials were recorded with Multiwell-Screen software (Multi Channel Systems) and analysed with Multiwell-Analyzer software (Multi Channel Systems). The data from the electrodes showing clear beating patterns, sodium spike voltage change over 70 µV, and a detectable T-wave were used for the analyses. Data obtained from the electrodes of one well were averaged. In the analyses, each well was treated as an individual and (the ratio of) test compound-induced changes were calculated against the recorded baseline from the same well. The data was normalized only against the baseline values, not against the vehicle control. All the field potential durations (FPD) were corrected against the changes in beating frequency using the Fridericia formula $cFPD=FPD/\sqrt[3]{RR}$, where RR is the RR interval.

For each compound, we analysed the effects on FPD, beat rate and amplitude. We selected one MEA parameter based on their mechanism of action. FPD, that corresponds to the QT interval^59^, was selected for compounds that are known to affect the QT time e.g., by targeting hERG or Ca^2+^ channels. Beat rate was selected for compounds that target adrenergic receptors. The strong transient spike in the MEA data results from Na + influx^59^, and the amplitude was selected for compounds targeting the Na^+^ channel.

### Quantification and statistical analysis

For the electrophysiology measurements, the chosen sample size was either 4 when using 24-well MEA plates with 12 electrodes per well and 8 when using 96-well MEA plates with 3 electrodes per well. The reported n stands for the number of wells (cardiac tissue models) per repetition of the study. Each test was repeated 3 times using 4–8 parallels in each concentration. Excel (Microsoft) was used for to calculate the p-values using Student’s two-tailed t-test. EC_50_ and IC_50_ values were calculated using non-linear fit four-parameters logistic equation Prism version 5.0. (GraphPad). The baseline values were used as E_min_. Because determination of E_max_ was shown to be case sensitive, the second plateau of the sigmoidal curve was taken as E_max_, except in case the plateau was not reached at the maximal concentration of 30 µM, the effect seen at this concentration was selected as E_max_. The data from electrodes that did not show clear beating patterns, sodium spike voltage changes over 70 µV and a detectable T-wave, were excluded from the analyses. On average approximately 15% of the wells had to be excluded from the experiment, if less than 3 wells out of 8 well per compound did pass the above-mentioned criteria the whole compound was excluded from the analysis of that experiment.

Gene expression (RNAseq) was performed for samples acquired from two replications the expression pattern of majority of the genes were similar for both replications according to sample clustering after principal component analysis (PCA). One sample was excluded from each timepoint for both culture types (cardiac tissue model and cardiomyocyte monoculture). The exclusion was done based on sample clustering after PCA. The excluded samples originated from the same cardiomyocyte culture batch. Benjamini–Hochberg procedure was applied for the data as described above.

Immunofluorescence and electrophysiology studies were replicated at least 3 valid times. Electron microscopy was performed once for each sample type with the 1–2 replicate wells indicating similar results. Independent samples Mann–Whitney U test was performed in IBM SPSS Statistics 27 to test the statistical significance of the cell and myofibril circular variances in monoculture and cardiomyocyte model.

## Supplementary Information

Supplementary Information 1.

Supplementary Information 2.

## Data Availability

A gene count matrix with corresponding metadata has been included in supplementary data.

## Code availability

Open-source and commercial codes and software that were used in this study are specified in the Methods. No custom codes or software were used.
