# Supplementary material for: Functional human cell-based vascularised cardiac tissue model for biomedical research and testing
Source: Sci Rep. 2022 Aug 5;12:13459. doi: 10.1038/s41598-022-17498-0 (PMC9355975; doi:10.1038/s41598-022-17498-0)
Supplement: Supplementary file 1 — Supplementary Information 1. [file 41598_2022_17498_MOESM1_ESM.pdf]

SUPPLEMENTARY MATERIAL

Functional human cell-based vascularised cardiac tissue model for biomedical research and testing

by Maria Koivisto, Tuomas A. Tolvanen, Tarja Toimela, Ilkka Miinalainen, Antti Kiviaho, Juha Kesseli, Matti Nykter, Lauri Eklund and Tuula Heinonen

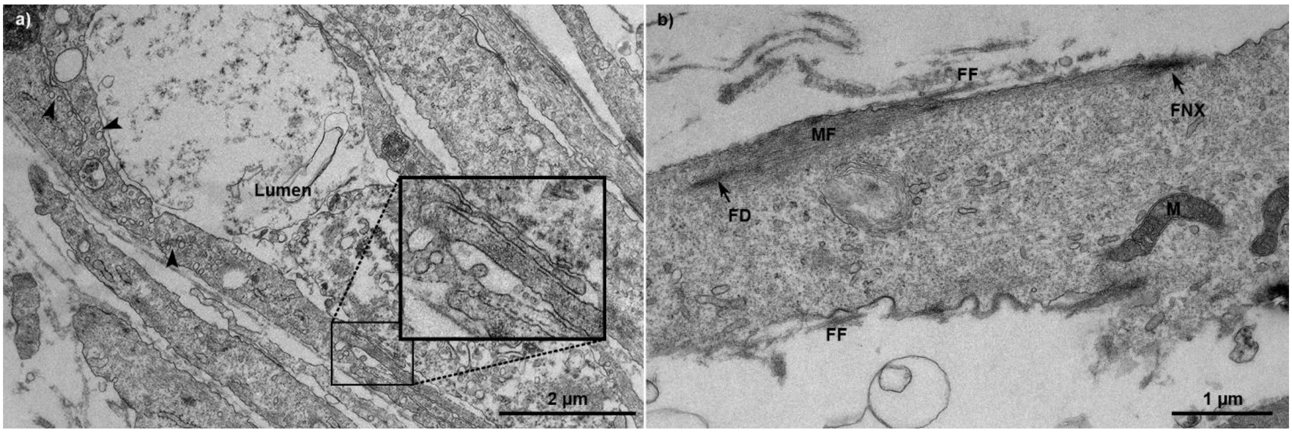

Supplementary Figure S1. Endothelial cells and myofibroblasts in cardiac tissue model. a) Endothelial cells are forming a vessel lumen in cardiac tissue model 1d after cardiomyocyte seeding. The arrow heads show transcytotic vesicles. Endothelial cells are characterized by large number of transcytotic vesicles. Endothelial cell-endothelial cell junctions are shown in the box (magnified). b) The myofibroblast in cardiac tissue model 6d after cardiomyocyte seeding. FD=focal density, FF=fibronectin filament, FNX=fibronexus, MF=myofilament, M=mitochondria. Scale bar 2 µm (a) and 1 µm (b).

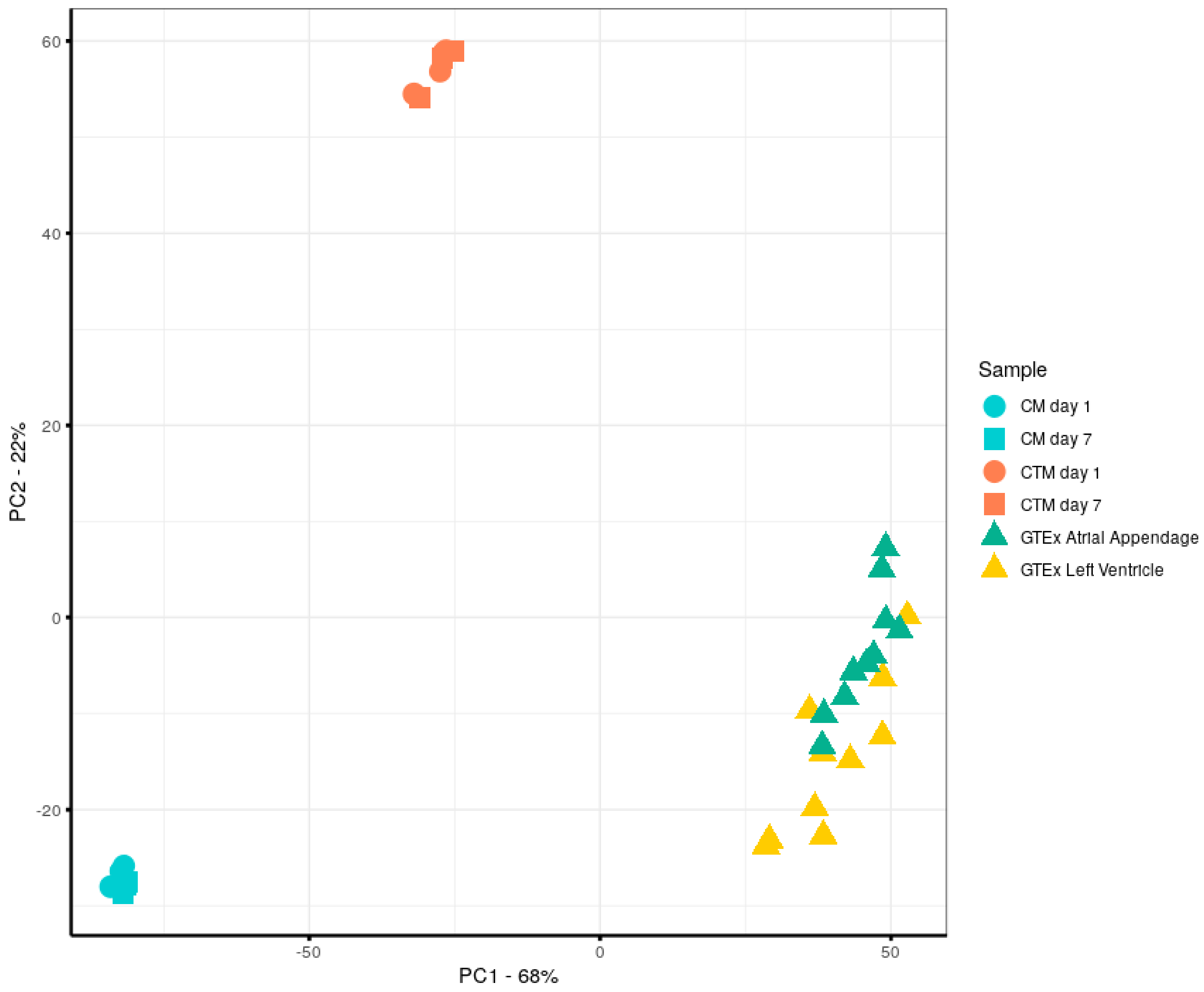

Supplementary Figure S2. PCA-plot with integrated mature heart tissue RNA sequencing data from the Genotype-Tissue Expression (GTEx) project as a positive control (Lonsdale, J. *et al.* The Genotype-Tissue Expression (GTEx) project. *Nat Genet* **45,** 580–585 (2013), https://doi.org/10.1038/ng.2653). These samples underwent the same preprocessing steps as our self-generated data, starting from count matrices. Gene expression patterns of the positive controls were found to be more similar to those of the cardiac tissue model along principal component 1, which attributes to the majority of inter-sample variance. Additionally, the 500 most variable genes used to perform PCA were found to be enriched with heart-specific genes previously identified using the same GTEx data (Ahn, J. *et al.* Integrative Analysis Revealing Human Heart-Specific Genes and Consolidating Heart-Related Phenotypes. *Frontiers in Genetics* **11**, (2020), https://www.frontiersin.org/article/10.3389/fgene.2020.00777) (Fisher's exact test, p=1.15e-10), highlighting the fact that PCA captures information from relevant genes.

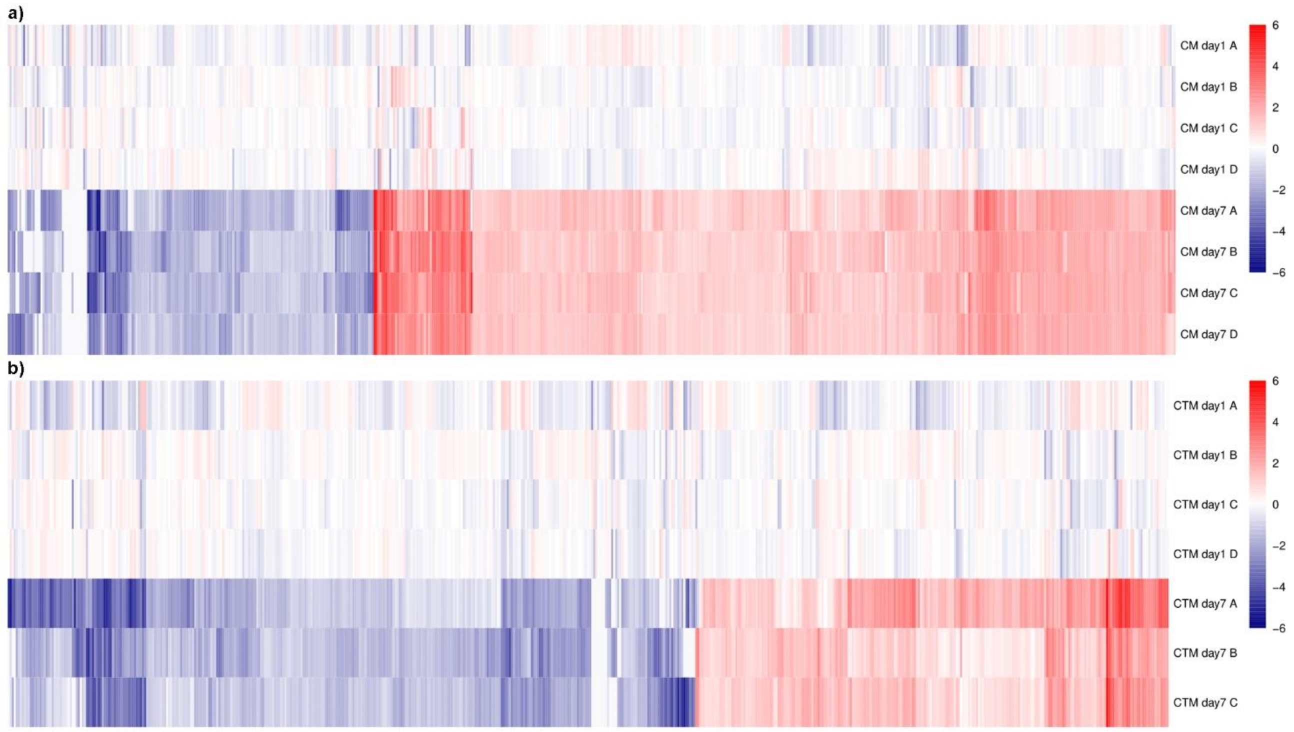

Supplementary Figure S3. Gene expression profiles in cardiomyocyte monocultures and cardiac tissue models. a) Heatmap of the log2-transformed fold changes (day 1 to day 7) of the cardiomyocyte monoculture samples for 604 differentially expressed genes. The DESeq2-normalized count of each gene was additionally normalized against the mean of the day 1 expression of each gene. b) Heatmap of the log2-transformed fold changes (day 1 to day 7) of the cardiac tissue model samples for 579 differentially expressed genes. The DESeq2-normalized count of each gene was additionally normalized against the mean of the day 1 expression of each gene. Both heatmaps were created in R 3.6.0 using pheatmap v1.0.12 (https://github.com/raivokolde/pheatmap)

A)

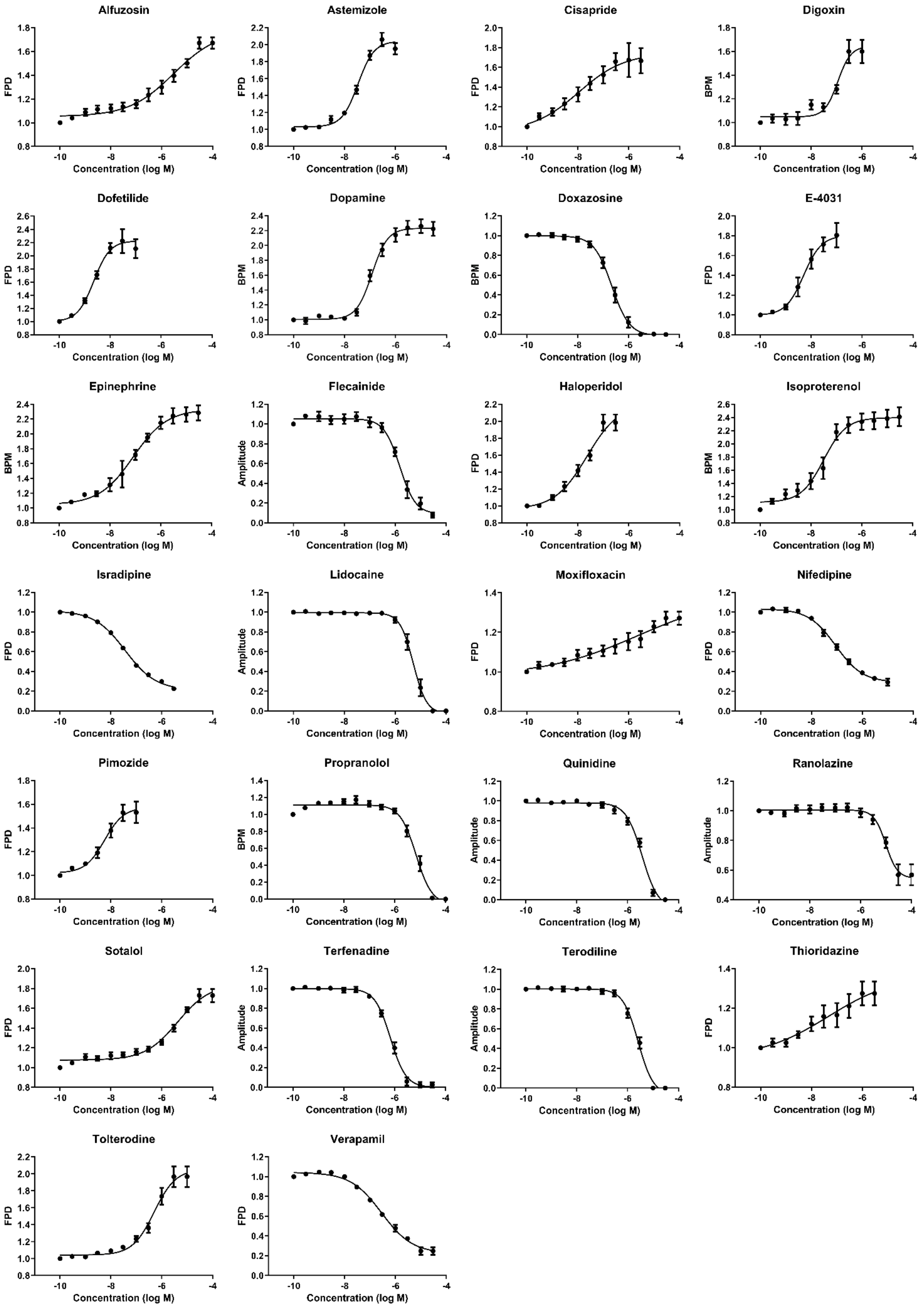
Alfuzosin
FPD
Concentration (log M)
Astemizole
FPD
Cisapride
FPD
Digoxin
BPM
Dofetilide
FPD
Dopamine
BPM
Doxazosine
BPM
E-4031
FPD
Epinephrine
BPM
Flecainide
Amplitude
Haloperidol
FPD
Isoproterenol
BPM
Isradipine
FPD
Lidocaine
Amplitude
Moxifloxacin
FPD
Nifedipine
FPD
Pimozide
FPD
Propranolol
BPM
Quinidine
Amplitude
Ranolazine
Amplitude
Sotalol
FPD
Terfenadine
Amplitude
Terodiline
Amplitude
Thioridazine
FPD
Tolterodine
FPD
Verapamil
FPD

B)

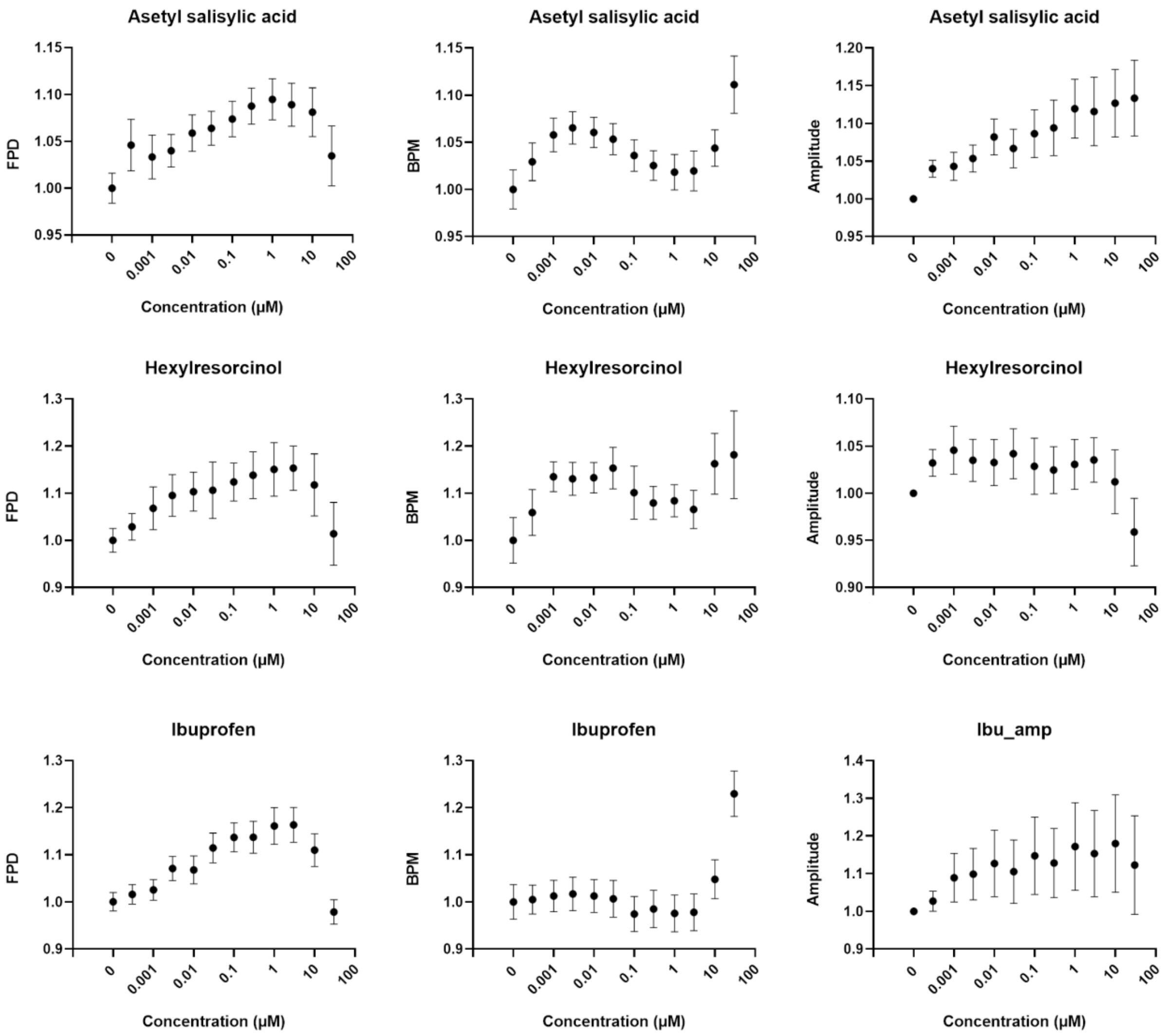

Supplementary Figure S4. A) Concentration-dependent effect of the study compounds on FPD, BPM or beating amplitude. The graphs have been used for EC_{50} calculations. B) Three negative controls did not have biologically significant effect on the heart function. Three independent repetitions, 3–8 parallels in each. Error bars represent standard error of the mean (SEM).

Supplementary Table S1. Lists of differentially expressed genes, enriched GO terms and enriched pathways.

(excel file)
